# Inhibition of Phosphodiesterase 4 by FCPR03 Alleviates Lipopolysaccharide-Induced Depressive-Like Behaviors in Mice: Involvement of p38 and JNK Signaling Pathways

**DOI:** 10.3390/ijms19020513

**Published:** 2018-02-08

**Authors:** Hui Yu, Zhengqiang Zou, Xiaolin Zhang, Wanli Peng, Chen Chen, Yicheng Ye, Jiangping Xu, Haitao Wang

**Affiliations:** 1 Department of Neuropharmacology and Drug Discovery, School of Pharmaceutical Sciences, Southern Medical University, Guangzhou 510515, China; yuhuiguangzhou@163.com (H.Y.); zhengqiangzou@163.com (Z.Z.); m18985616955_1@163.com (X.Z.); pwl178@163.com (W.P.); m15626426587_1@163.com (C.C.); ycheng0713@163.com (Y.Y.); wht821@smu.edu.cn (H.W.); 2 Guangdong Provincial Key Laboratory of New Drug Screening, School of Pharmaceutical Sciences, Southern Medical University, Guangzhou 510515, China; 3 Department of Pharmacy Intravenous Admixture Service, Ganzhou People’s Hospital, Ganzhou 341000, China

**Keywords:** phosphodiesterase 4, FCPR03, depression, p38, JNK

## Abstract

Inflammatory responses induced by peripheral administration of lipopolysaccharide (LPS) triggers depressive-like behavioral syndrome in rodents. Inhibition of phosphodiesterase 4 (PDE4) produces a robust anti-inflammatory effect in inflammatory cells. Unfortunately, archetypal PDE4 inhibitors cause intolerable gastrointestinal side-effects, such as vomiting and nausea. *N*-isopropyl-3-(cyclopropylmethoxy)-4-difluoromethoxy benzamide (FCPR03) is a novel, selective PDE4 inhibitor with little, or no, emetic potency. Our previous studies show that FCPR03 is effective in attenuating neuroinflammation in mice treated with LPS. However, whether FCPR03 could exert antidepressant-like effect induced by LPS is largely unknown. In the present study, mice injected intraperitoneally (i.p.) with LPS was established as an in vivo animal model of depression. The antidepressant-like activities of FCPR03 were evaluated using a tail suspension test, forced swimming test, and sucrose preference test. We demonstrated that administration of FCPR03 (1 mg/kg) produced antidepressant-like effects in mice challenged by LPS, as evidenced by decreases in the duration of immobility in the forced swim and tail suspension tests, while no significant changes in locomotor activity were observed. FCPR03 also increased sucrose preference in mice treated with LPS. In addition, treatment with FCPR03 abolished the downregulation of brain-derived neurotrophic factor induced by LPS and decreased the level of corticosterone in plasma. Meanwhile, periphery immune challenge by LPS induced enhanced phosphorylation of p38-mitogen activated protein kinase (p38) and c-Jun N-terminal kinase (JNK) in both the cerebral cortex and hippocampus in mice. Interestingly, treatment with FCPR03 significantly blocked the role of LPS and reduced the levels of phosphorylated p38 and JNK. Collectively, these results indicate that FCPR03 shows antidepressant-like effects in mice challenged by LPS, and the p38/JNK signaling pathway is possibly involved in this process. Our findings suggest that FCPR03 is a potential compound for the prevention or treatment of depression.

## 1. Introduction

Depression is a common and debilitating neuropsychiatric disorder with increasing incidence year by year. Currently, depression is considered to be one of the major global mental health problems, as self-harm events and suicide attempts usually occur in patients diagnosed with this disorder [1]. The etiology of depression is complicated and multiple factors have been proved to be involved in the pathology of this disease [2]. Among multiple hypotheses, deficiency of monoamine transmitters in the synaptic cleft is the most acceptable explanation for this disorder [3], and most of the commercially-available drugs are developed based on this theory [3]. Current antidepressants, such as serotonin reuptake inhibitors, are effective in alleviating the clinical symptoms of depression through enhancing the level of neurotransmitters in the synaptic cleft. However, there are also many limitations for these drugs. For example, 30–40% of treated patients unsuccessfully respond to current antidepressants [2], especially patients with moderately depressed syndromes [4]. Additionally, nervousness, insomnia, and sexual dysfunction associated with these drugs are still unable to be tolerated in some patients [5,6]. Moreover, current antidepressants have a delayed onset of action, and most of the drugs take several weeks to reach efficacy [7]. Due to these limitations, a totally novel target and new class of antidepressant agents urgently needs to be developed.

The cytokine hypothesis of depression emphasizes that internal or external stress induces cytokine imbalances which trigger the expression and continuity of depressive symptoms in vulnerable individuals [8]. Accumulating evidence reveals neuroinflammation is closely associated with the pathogenesis of depression. The levels of several inflammatory markers, including tumor necrosis factor-α (TNF-α), interleukin-1β (IL-1β), and interleukin-6 (IL-6), are significantly increased in patients with depression compared to these in normal subjects [9], while administration of IL-1 receptor antagonist (IL-1ra) ameliorated depressive-like behaviors [10]. Lentivirus-mediated IL-1β knock-down in the hippocampus also alleviates lipopolysaccharide (LPS)-induced depressive-like behaviors in mice [11]. Based on the neuroinflammation hypothesis of depression, LPS-treated mice are widely used to study the underlying mechanisms of depression and evaluate the efficiency of potential antidepressants [12,13,14]. Binding of LPS to its cognate receptors, such as CD14 and Toll-like receptors in mammalian cell membranes, activates several signaling cascades, such as p38-mitogen activated protein kinase (p38) and c-Jun N-terminal kinase (JNK) signaling pathways, and thereby regulates various genes encoding inflammatory mediators [15,16]. As downstream effectors of LPS stimulation, both JNK and p38 have been linked to the development of depression, the levels of phosphorylated JNK and p38 in the hippocampi of mice subjected to chronic, unpredictable, mild stress are higher than that in the wild-type control group mice [17]. Consistently, in neuronal-like cells, LPS robustly stimulated the phosphorylation of JNK and p38, while antidepressant trazodone effectively counteracted inflammatory signaling and decreased the elevation of phosphorylated JNK and p38 [18]. Overall, current studies support that inflammatory cytokines or exposure to psychologically-acute stressors induces p38/JNK activation in the brain, and the signaling pathways of pro-inflammatory cytokines contribute to the pathogenesis of depression.

As inflammatory responses are thought to contribute to the development of depression [9,10], anti-inflammatory drugs are expected to be effective in alleviating depressive-like behaviors. Phosphodiesterase 4 (PDE4) is an enzyme predominantly responsible for the hydrolysis of the second messenger cyclic adenosine monophosphate (cAMP) within both immune cells and in the central nervous system (CNS) [19]. PDE4 inhibitors have been widely used for the treatment of inflammatory diseases since the 1980s [20]. In the clinic, PDE4 inhibitors, such as roflumilast and apremilast, are mainly used for the treatment of chronic obstructive pulmonary disease, asthma, and psoriasis [21]. Inhibition of PDE4 leads to elevated level of intracellular cAMP, which subsequently activates protein kinase A (PKA). Activated PKA inhibits the production of nitric oxide and reduces the expression of inducible nitric oxide synthase (iNOS) and TNF-α, These effects are associated with a decrease in nuclear factor-κB (NF-κB) p65 DNA binding [22]. Hence, inhibition of PDE4 leads to decreased levels of pro-inflammatory factors through a cAMP-, PKA-, and NF-κB-dependent mechanism [23]. On the other hand, PDE4 inhibitors may also promote the transcription of anti-inflammatory cytokine IL-10 through the PKA/cAMP-response element binding protein (CREB) signaling pathway [23]. In the CNS, an increasing number of studies have shown that PDE4 plays a key role in neurodegenerative diseases (e.g., Alzheimer’s disease), mental disorders (such as depression and schizophrenia), and stroke as well [24,25,26]. Inhibition of PDE4 in the CNS contributes to the production of brain-derived neurotrophic factor (BDNF) and decreased expression of apoptosis-related proteins, such as cleaved caspase 3 [27,28]. BDNF is a member of the neurotrophin family of growth factors and the neurotrophic factors hypothesis of depression is nowadays widely acknowledge [29]. The level of BDNF is decreased in both the serum and brain during the course of major depression [30], while successful pharmacological antidepressant treatment is effective to recover the level of BDNF to baseline levels in untreated patients suffering from major depression [31]. Therefore, compounds enhanced the level of BDNF are supposed to be helpful for the treatment of depression. Collectively, inhibition of PDE4 is a promising strategy to treat both neurological and psychiatric diseases. However, dose limiting side effects of nausea, diarrhea and headache have tempered the enthusiasm of this drug class for the treatment of these diseases [32,33]. A number of strategies are currently being pursued in attempts to improve clinical efficacy and reduce side effects, including development of non-emetic PDE4 inhibitors.

FCPR03 is a novel selective PDE4 inhibitor with IC_50_ of 60 nM, 31 nM, and 47 nM for the core catalytic domains of PDE4, PDE4B, and PDE4D, respectively. This compound has no inhibitory effects on other PDE isoforms [32]. Our previous studies found that FCPR03 inhibited LPS-induced expression of TNF-α, IL-1β, and iNOS through activating the cAMP/PKA/CREB signaling pathway and inhibition of NF-κB in microglial cells [32,33]. Importantly, compared with the prototype PDE4 inhibitor rolipram, FCPR03 did not cause emesis in beagle dogs at the effective dose [32], suggesting that FCPR03 is a novel PDE4 inhibitor with little or no emetic potency. In LPS-challenged mice, we also found that FCPR03 significantly ameliorated the loss of appetite and cognitive deficits. However, whether FCPR03 is effective to reverse LPS-triggered depressive-like behaviors is still unknown. In the present study, the effects of FCPR03 in LPS-induced depressive-like behaviors were evaluated. Furthermore, whether JNK and p38 signaling pathways are involved in the anti-depressant-like effects of this compound were assessed.

## 2. Results

### 2.1. Effect of LPS on Body Weight and Immobility Time in C57BL/6 Mice

**Figure 1:**
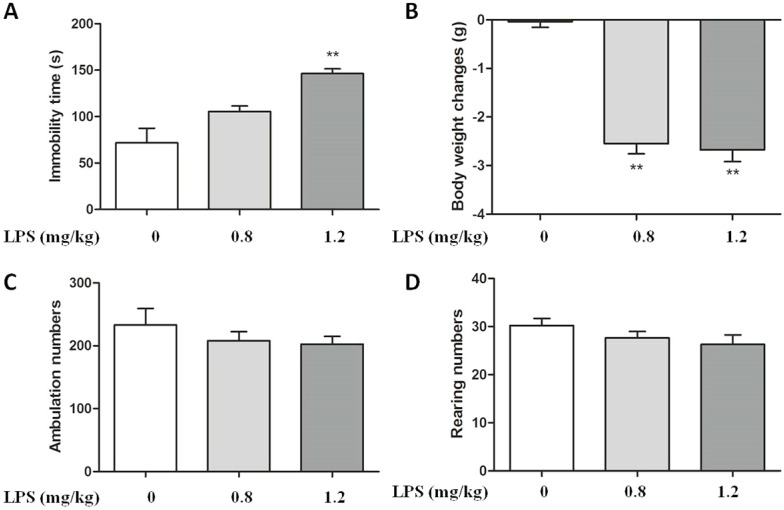
LPS induced depressive-like behavior in C57BL/6 mice. Mice were injected i.p. with 0.8 mg/kg or 1.2 mg/kg LPS, 24 h after LPS injection, immobility time in the forced swimming test (**A**), body weight (**B**), the numbers of ambulation in the open field test (**C**), and the numbers of rearing in the open field test (**D**) were measured. Data are expressed as the mean ± SEM, ** *p* < 0.01 compared with control group (*n* = 8 per group).

In order to investigate the antidepressant role of FCPR03 in mice, firstly, depressive behaviors were mimicked in C57BL/6 mice by intraperitoneal (i.p.) injection of LPS at dosages of 0.8 and 1.2 mg/kg. Twenty-four hours after the injection of LPS, a forced swimming test was performed to assess the behavioral despair. As shown in Figure 1A, treatment with LPS in C57BL/6 mice significantly reduced the immobility time in forced swimming test at the dosage of 1.2 mg/kg (*p* < 0.01) (Figure 1A), we thus selected this concentration for subsequent studies. We also found that injection of LPS at doses of 0.8 mg/kg and 1.2 mg/kg reduced the body weight in mice (*p* < 0.01) (Figure 1B). In order to test whether or not the data of immobility time evaluated in the forced swimming test could be associated with a change of the spontaneous locomotor activity of mice. LPS was tested alone in the locomotor activity. The results of locomotor activity was shown in Figure 1C,D, Statistical analysis by one-way analysis of variance (ANOVA) showed no significant difference in the open field test (*p* > 0.05). Hence, these data suggest that LPS injection has no effect on spontaneous locomotor activity, while LPS induces depressive-like behaviors and decreases body weight in mice.

### 2.2. The Antidepressant Effect of FCPR03 in LPS-Treated Mice

**Figure 2:**
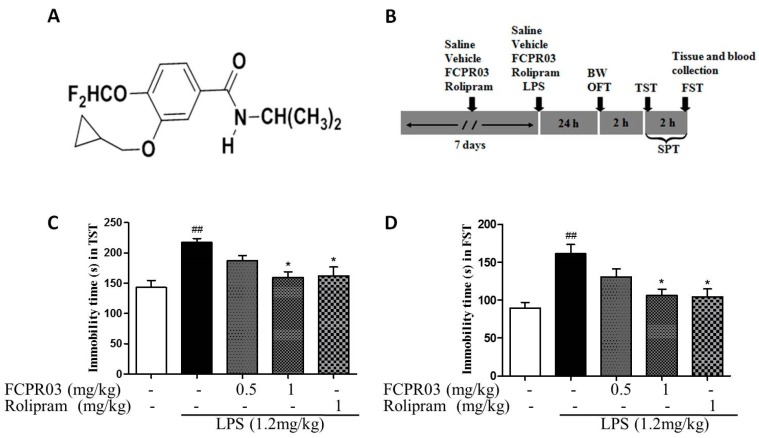
Effect of pre-treatment of mice with FCPR03 or rolipram on the LPS-induced reductions in the immobility time in the tail suspension test and forced swimming test. The chemical structure of FCPR03 (**A**) and experimental time line (**B**). After seven consecutive days of pretreatment with FCPR03 (0.5 mg/kg or 1 mg/kg) or rolipram (1 mg/kg), mice were injected i.p. with saline or LPS (1.2 mg/kg, i.p.); 24 h after LPS injection, the immobility time in tail suspension test (**C**) and forced swimming test (**D**) were measured. Data are expressed as the mean ± SEM, ^##^ *p* < 0.01 compared with control group; * *p* < 0.05 compared with LPS group (*n* = 8 per group). BW, body weight; OFT, open field test; TST, tail suspension test; FST, forced swimming test; SPT, sucrose preference test.

Having established depressive-like behaviors in mice treated with LPS, we next investigated the antidepressant-like effect of FCPR03. The chemical structure of FCPR03 is shown in Figure 2A and the experimental timeline is shown in Figure 2B. Both forced swimming test and tail suspension test were used for evaluating antidepressant effect of FCPR03. Rolipram, a canonical PDE4 inhibitor, was used as a positive control. As shown in Figure 2C,D, compared with mice in control group, mice treated with LPS showed a significant increase in immobility time in both forced swimming test and tail suspension test (*p* < 0.01). As expected, 1 mg/kg FCPR03 attenuated the role of LPS and decreased the immobility time (*p* < 0.05), suggesting that FCPR03 possesses antidepressant-like effects. The positive control rolipram (1 mg/kg) also showed a significant antidepressant effect (Figure 2C,D).

**Figure 3:**
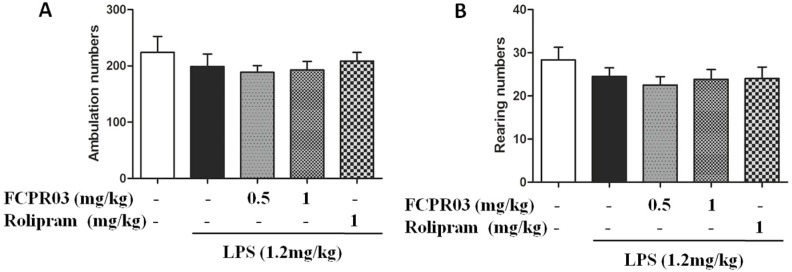
Effect of treatment of mice with FCPR03 or rolipram combined with LPS in the open field test. After seven consecutive days of pretreatment with FCPR03 (0.5 mg/kg or 1 mg/kg) or rolipram (1 mg/kg), mice were injected i.p. with saline or LPS (1.2 mg/kg, i.p.); 24 h after LPS injection, the ambulation activity (**A**) and rearing activity (**B**) were measured (*n* = 8 per group).

In order to rule out an influence of the locomotor activity elicited by the treatments on the immobility time in the forced swimming test and tail suspension test, the open field test was conducted. Figure 3 shows the effect of LPS (1.2 mg/kg) combined with FCPR03 (0.5 mg/kg or 1 mg/kg) in the open field test. This combined treatment did not produce change in the locomotor activity of mice (Figure 3A,B), since the results did not show any significant differences among the control, LPS, and FCPR03 combined with LPS groups (*p* > 0.05).

### 2.3. FCPR03 Increased Sucrose Preference in Mice Challenged with LPS

**Figure 4:**
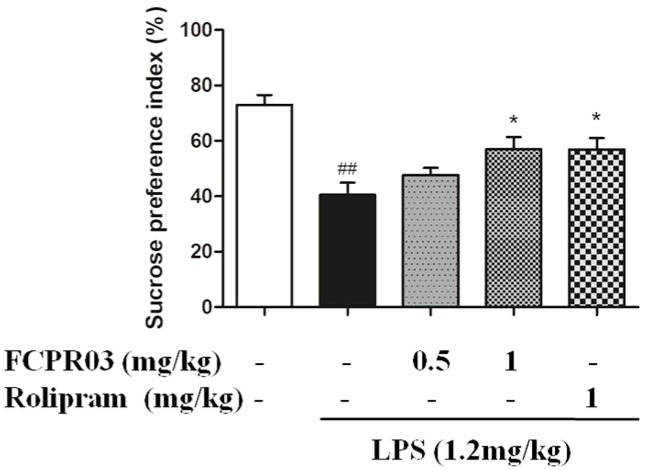
FCPR03 increased sucrose preference in mice challenged with LPS during sucrose intake test. After seven consecutive days of pretreatment with FCPR03 (0.5 mg/kg or 1 mg/kg) or rolipram (1 mg/kg), mice were injected i.p. with saline or LPS (1.2 mg/kg, i.p.); 24 h after LPS injection, the preference to sucrose solution were measured. Data are expressed as the mean ± SEM. ^##^ *p* < 0.01 compared with control group; * *p* < 0.05 compared with LPS group (*n* = 8 per group).

The core symptom of depression is loss of interest, which could be assessed by sucrose preference in rodents [34]. In the present study, sucrose preference is calculated as a percentage of the volume of sucrose intake over the total volume of fluid intake. As shown in Figure 4, one-way ANOVA showed significant differences among experimental groups. The preference to sucrose solution was significantly decreased in the LPS-challenged mice compared with that in mice in the control group (*p* < 0.01), confirming behavioral depression in these mice, while treatment with FCPR03 (1 mg/kg) and rolipram (1 mg/kg) restored the sucrose preference in mice treated with LPS (*p* < 0.05).

### 2.4. FCPR03 Decreased the Level of Plasma Corticosterone in LPS-Treated Mice

**Figure 5:**
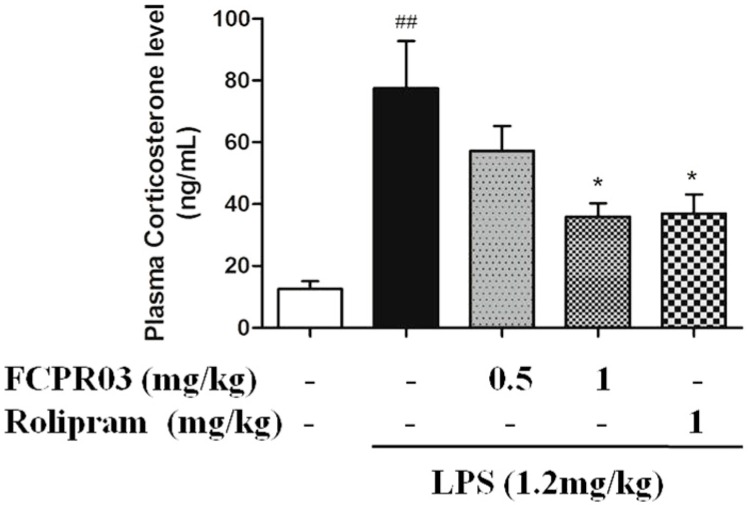
FCPR03 decreased the level of plasma corticosterone in mice challenged with LPS. After seven consecutive days of pretreatment with FCPR03 (0.5 mg/kg or 1 mg/kg) or rolipram (1 mg/kg), mice were injected i.p. with saline or LPS (1.2 mg/kg, i.p.); 24 h after LPS injection, the level of plasma corticosterone were measured by ELISA. Data are expressed as the mean ± SEM. ^##^ *p* < 0.01 compared with control group; * *p* < 0.05 compared with LPS group (*n* = 8 per group).

Acute or chronic stress results in hyperactivity of the hypothalamic-pituitary-adrenal (HPA) axis, which will lead to enhanced vulnerability to depression, and the mechanisms are supposed to be associated with abnormalities in neurogenesis, neuroplasticity, and/or neurotoxicity [35,36,37], particularly in the hippocampus, which is involved in the development of major depression [36]. Hence, in the present study, we investigated the role of FCPR03 on the level of plasma corticosterone in LPS-treated mice. As shown in Figure 5, LPS-treated mice showed significant elevation in their serum level of corticosterone in comparison to the control group (*p* < 0.01). While treatment with FCPR03 (1 mg/kg) showed a significant decrease of the serum level of corticosterone (*p* < 0.05), the role of FCPR03 on the level of corticosterone was similar to rolipram (1 mg/kg).

### 2.5. FCPR03 Stimulated the Expression of BDNF in LPS-Treated Mice

**Figure 6:**
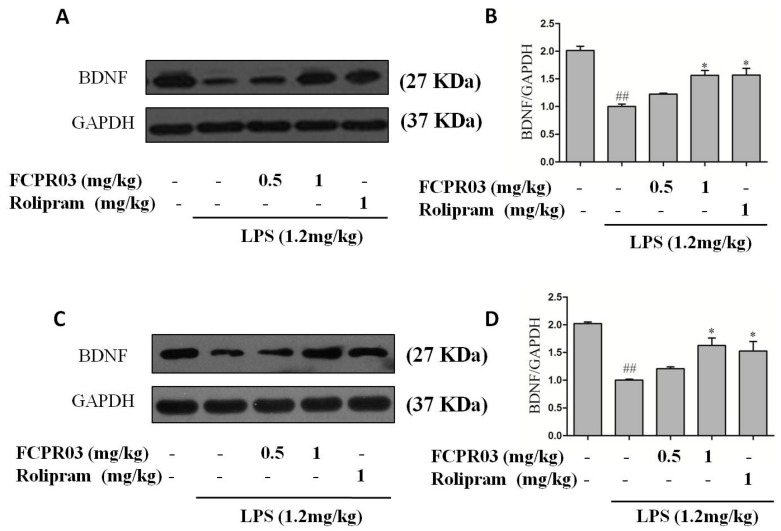
FCPR03 enhanced the level of BDNF in both cortex and hippocampus in mice challenged with LPS. After seven consecutive days of pretreatment with FCPR03 (0.5 mg/kg or 1 mg/kg) or rolipram (1 mg/kg), mice were injected i.p. with saline or LPS (1.2 mg/kg, i.p.); 24 h after LPS injection, the entire hippocampal and cortical extracts were homogenized, and Western blot was used to assess BDNF protein levels in the cortex (**A**) and hippocampus (**C**). The corresponding quantification data are shown in (**B**,**D**). Data are expressed as the mean ± SEM. ^##^ *p* < 0.01 compared with control group; * *p* < 0.05 compared with LPS-treated group (*n* = 8 per group).

Our previous data revealed that FCPR03 possessed a potent anti-inflammatory property in both in vitro and in vivo inflammatory models through regulating the cAMP/PKA/CREB signaling pathway [33]. BDNF is the most widely-distributed neurotrophic factor in the central nervous system and, importantly, activated CREB directly regulates the transcription of BDNF [38]. Increased expression of BDNF in the hippocampus and cortex produces an antidepressant-like effect in both corticosterone and chronic unpredictable mild stress-induced depression-like animal models [39]. In order to investigate the effect of FCPR03 on the expression of BDNF, LPS-treated mice were pretreated with 0.5 and 1 mg/kg FCPR03, and BDNF was detected by Western blot. We found that LPS suppressed the expression of BDNF in both cortical (*p* < 0.01) (Figure 6A,B) and hippocampal (*p* < 0.01) (Figure 6C,D) tissues in mice. Treatment with FCPR03 at 1 mg/kg significantly increased the protein expression of BDNF (*p* < 0.05) in these brain region in LPS-treated mice, rolipram at 1 mg/kg exhibited a similar effect to FCPR03 (1 mg/kg) on the expression of BDNF.

### 2.6. FCPR03 Increased the Phosphorylation of p38 and JNK in LPS-Treated Mice

**Figure 7:**
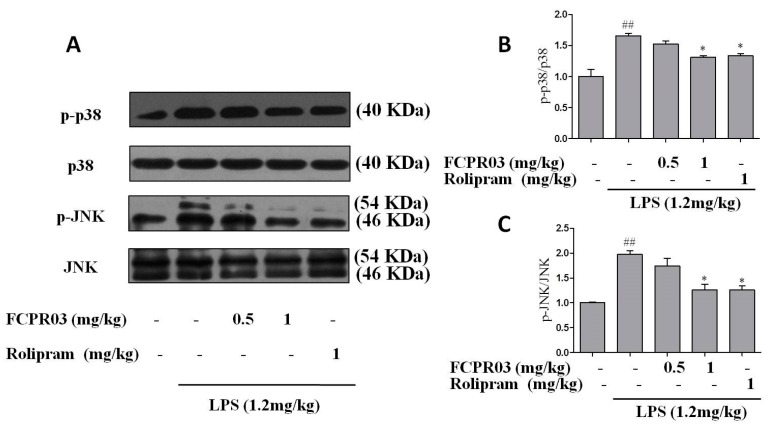
FCPR03 attenuated the phosphorylation of p38 and JNK in cortex in mice challenged with LPS. After seven consecutive days of pretreatment with FCPR03 (0.5 mg/kg or 1 mg/kg) or rolipram (1 mg/kg), mice were injected i.p. with saline or LPS (1.2 mg/kg, i.p.); 24 h after LPS injection, the entire cortical extracts were homogenized, and Western blot was used to assess the phosphorylated p38 (p-p38), total p38, phosphorylated JNK (p-JNK), and total JNK protein levels in the cortex (**A**). The corresponding quantification data are shown in (**B**,**C**). Data are expressed as the mean ± SEM. ^##^ *p* < 0.01 compared with control group; * *p* < 0.05 compared with LPS-treated group (*n* = 8 per group).

**Figure 8:**
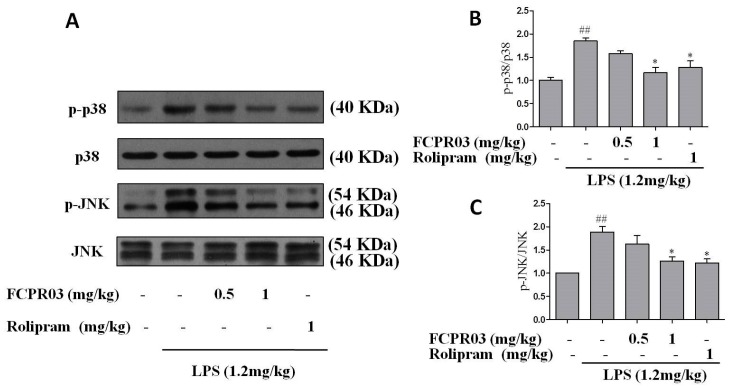
FCPR03 attenuated the phosphorylation of p38 and JNK in hippocampus in mice challenged with LPS. After seven consecutive days of pretreatment with FCPR03 (0.5 mg/kg or 1 mg/kg) or rolipram (1 mg/kg), mice were injected i.p. with saline or LPS (1.2 mg/kg, i.p.); 24 h after LPS injection, the entire hippocampal extracts were homogenized, and Western blot was used to assess the phosphorylated p38 (p-p38), total p38, phosphorylated JNK (p-JNK), and total JNK protein levels in the cortex (**A**). The corresponding quantification data are shown in (**B**,**C**). Data are expressed as the mean ± SEM. ^##^ *p* < 0.01 compared with control group; * *p* < 0.05 compared with LPS-treated group (*n* = 8 per group).

JNK and p38 signal pathways were closely related with inflammatory reaction and depression [15,18,40]. Our previous data also indicated that FCPR03 had anti-inflammatory properties in LPS-treated mice [33]. So far, we have shown the anti-depressant-like effect of FCPR03 in mice model. To further explore whether p38 and JNK signaling pathways are involved in the anti-depressant-like effect of FCPR03, Western blot was used to determine the expression of phosphorylated p38 and JNK in both the cortical and hippocampal tissues. As shown in Figure 7 and Figure 8, compared with mice in the control group, the phosphorylation of p38 and JNK in the cortex (Figure 7A–C) and hippocampus (Figure 8A–C) were increased in mice treated with LPS (*p* < 0.01). Treatment with FCPR03 at 1 mg/kg, as well as rolipram at 1 mg/kg, blocked the phosphorylation of p38 (*p* < 0.05) and JNK (*p* < 0.05). These results demonstrate that p38 and JNK are activated following LPS administration, while treatment with FCPR03 decreases the phosphorylation of both p38 and JNK and, thus, reduced activity of p38 and JNK.

## 3. Discussion

In this study, we show, for the first time, that selective inhibition of PDE4 by FCPR03 attenuates LPS-induced depressive-like behaviors in mice. This is based on the following observations: (1) treatment with FCPR03 increased the immobility time in both the forced swimming test and tail suspension test, while FCPR03 had no significant effect in total locomotor activity; (2) FCPR03 significantly increased the sucrose preference in mice; (3) inhibition of PDE4 by FCPR03 resulted in reduced activation of hypothalamic-pituitary-adrenal (HPA) axis and decreased level of corticosterone; (4) FCPR03 promoted the expression of BDNF in both cortex and hippocampus; and (5) LPS stimulated the phosphorylation of JNK and p38, while FCPR03 blocked the role of LPS and suppressed the phosphorylation of JNK and p38 in the cortex and hippocampus of mice subjected to LPS. In the present study, our presented data also showed that the antidepressant effect of FCPR03 was similar to the canonical PDE4 inhibitor rolipram. Unfortunately, rolipram induces vomiting at doses comparable to, or less than, its efficacious doses; the clinical application of rolipram has been hindered by the intolerable side effects [19]. In contrast, FCPR03 is a novel PDE4 inhibitor, and it has no effect in inducing emesis [29]. Hence, FCPR03 has obvious advantages concerning the side effects and drug safety.

We previously reported that acute administration of FCPR03 produced antidepressant-like effects [32]. Since elevated pro-inflammatory factors are observed in both mouse models of depression and patients diagnosed with major depression, we induced chronic neuroinflammation by injecting LPS in the current study. Our previous studies also suggested that mice injected with LPS showed an over-production of IL-1β, TNF-α, and IL-6 [33]. In the present study, we successfully showed that FCPR03 also ameliorated LPS-induced depressive-like behaviors. These results support our suggestion that FCPR03 functions as an effective antidepressant drug, as evidenced by decreases in the duration of immobility in the forced swimming test and tail suspension test, and increased sucrose preference in mice.

The continuous activation of the HPA axis plays a stimulatory effect in the pathology of depression [4]. Chronic activation of the HPA axis promotes the secretion of adrenocorticotrophic hormone from the pituitary gland, which ultimately leads to the abnormally-increased levels of glucocorticoids from the adrenal cortex [4]. Excessive glucocorticoids in the brain impairs hippocampal granule cells and increases susceptibility to various stressful situations, including depression [37,41]. In the present study, the endocrine changes in the HPA axis were observed following LPS administration. Our results showed that FCPR03 treatment caused a significant reduction in plasma corticosterone induced by LPS. These data strongly suggested that FCPR03 exerted antidepressant activity, at least in part, by regulating the corticosterone level, thus normalizing the HPA axis hyperactivity. Of note, the role of PDE4 inhibitors on HPA axis activity and the level of corticosterone in serum/plasma are not consistent in different animal models. In basal conditions, the PDE4 inhibitor was reported to increase the basal HPA axis activity, and increase the plasma concentrations of corticotrophin and corticosterone 30 min post injection [42]. Administration of the PDE4 inhibitor denbufylline (0.1–2.5 mg/kg, i.p.) produced a significant increase in the serum corticosterone concentration in adult male rats [43,44]. These results were supported by the findings that intraperitoneal administration of rolipram (1–30 mg/kg) caused a dose-dependent increase in the circulating level of corticosterone in male Balb/c mice [45]. However, when denbufylline was administered by intracerebroventricular injection, it failed to affect the serum corticosterone concentration [43,44]. In a traumatic brain injury animal model, no significant change was observed in the serum corticosterone level after treatment with PDE4 inhibitor etazolate [46]. Consistent with what we found in the study, studies also showed that PDE4 inhibitors significantly decreased the serum corticosterone in different animal models of depression. For example, Wang et al., reported that animals exposed to the chronic unpredictable stress, a rodent model of depression, exhibited elevated corticosterone, while the increased level of corticosterone was reversed by the PDE4 subtype nonselective inhibitor rolipram [47]. Consistently, in another olfactory bulbectomy-induced depression animal model, rolipram significantly decreased the corticosterone level in animals [48]. The discrepancy may be attributed to the different experimental design, species, different animal model, and the dosing of the inhibitors. As corticosterone is raised in LPS-treated mice in the present study, it is not surprising that there was a decreased BDNF level in the hippocampal and cortical tissues of LPS-treated mice as compared to the control group. An elevated level of the corticosterone has emerged recently as one of the factors contributing to the pathogenesis of depression [4], and the role of corticosterone is probably mediated through causing specific structural and functional deficits in the hippocampus, a major center for emotional processing and stress-induced behaviors [49]. Dysfunction of hippocampus caused by HPA hyperactivity leads to reduced expression of BDNF. Chronic administration of antidepressant drugs have been proved to increase neurogenesis and enhance restoration of neuronal plasticity in animal models of depression [50,51]. In accordance with these findings, our data shows that LPS induced the down-regulation of BDNF in the hippocampus and cortex, while FCPR03 effectively increased the level of BDNF in these two brain regions. In the present study, we also found that rolipram produced a similar effect with FCPR03, as rolipram induces emesis in rodents, while FCPR03 has no or at least less potential to induce vomiting and emesis [32]. Hence, FCPR03 has more advantages to be developed as a candidate antidepressant drug.

FCPR03 is a selective PDE4 inhibitor, inhibition of PDE4 by FCPR03 activates the cAMP/ PKA/CREB signaling pathway [33]. Our previous studies also showed that silencing PDE4 by shRNA, or inhibiting PDE4 activity through highly-specific inhibitors, significantly increased the level of cAMP and the phosphorylation of CREB in the brain [22,52]. BDNF is a direct target of CREB, and activated CREB binds to the promoter region of BDNF and regulates its transcription [53]. Increased BDNF has been proved to promote neuronal plasticity, which is beneficial for the alleviation of depressive-like behaviors in depressed patients [54]. On the other hand, treatment with PDE4 inhibitors is supposed to increase the activation of BDNF-TrkB signaling, hence, this signaling pathway is useful for screening novel antidepressant drugs. Results obtained in the present studies also support the neurotrophic factors hypothesis of depression. Hence, we propose that FCPR03 exerts antidepressant-like effects in mice, at least in part, by increasing the expression of BDNF in the brain, and this role is possibly mediated via the induction of the cAMP/PKA/CREB signaling pathway. The JNK and p38 MAPK signaling pathways are activated by multiple types of cellular stress including proinflammatory cytokines, such as TNF-α and IL-1β [55,56]. Stimulation of microglia with LPS shows activation of both JNK and p38MAPK and, thus, play key roles in increased inflammatory mediators like iNOS, IL-1β, or TNF-α [57,58]. On the other hand, both p38 and JNK signal pathways are activated and involved in pathophysiological changes in rat models of depression [59]. Given the central role of p38 and JNK signaling pathways in the regulation of neuroinflammation and depression, further studies are required to determine the relationship between the cAMP/PKA/CREB/BDNF and p38/JNK signaling pathways and their relative roles in LPS-induced depressive-like behaviors.

In summary, in the present study, we studied the antidepressant role of a novel PDE4 inhibitor FCPR03 in LPS-treated mice. FCPR03 reduced immobility time in the FST and TST, and reversed LPS-induced deficit in sucrose intake to prevent anhedonia in mice. Moreover, FCPR03 modified neuroendocrine activity by significantly reducing the plasma corticosterone level in LPS-challenged animal models. These results suggest that FCPR03 possess potent antidepressant properties and, potentially, JNK and p38 signaling pathways are involved in this effect.

## 4. Materials and Animals

### 4.1. Animals

Adult male C57BL/6 mice (22–25 g) were obtained from the Laboratory Animal Center of Southern Medical University (Guangzhou, China). Animals were housed at 22 ± 1 °C with a 12 h:12 h light/dark cycle. Mice had free to access food and water, and were acclimated in the facility for seven days before the experiments. All experimental procedures were carried out in accordance with the NIH Guide for the Care and Use of Laboratory Animals and approved by the Animal Care and Use Ethics Committee of the Southern Medical University (Resolution No. L2015053, date of resolution: 21 September 2015).

### 4.2. Drug Preparation and Treatment

Lipopolysaccharide (LPS, from *Escherichia coli* strain 055:B5) was purchased from Sigma-Aldrich Corp. (St. Louis, MO, USA), rolipram was purchased from the Enzo Life Sciences (Farmingdale, NY, USA). FCPR03 or *N*-isopropyl-3-(cyclopropylmethoxy)-4-difluoromethoxy benzamide (Figure 2A), a selective PDE4 inhibitor, was synthesized by Neuropharmacology and Drug Discovery Group, Southern Medical University (Guangzhou, China) [32]. Before administration, FCPR03 and rolipram were diluted with the vehicle (0.5% dimethylsulfoxide (DMSO), 0.5% carboxymethylcellulose sodium) to obtain working solutions. The drugs were prepared freshly before use. All other chemicals used were of analytical grade.

### 4.3. Mouse Model of Depression

To assess the LPS-induced behavioral changes, all mice were randomly divided into three experimental groups (*n* = 8), control, and LPS (LPS 0.8 mg/kg and 1.2 mg/kg, respectively). LPS was administered intraperitoneally (i.p.) to induce depressive-like behavior in mice, as previously described [60], and the behavioral experiments were performed 24 h after the administration of LPS. To explore the antidepressant-like effect of FCPR03, 40 mice were randomly divided into five groups, and received vehicle, LPS (1.2 mg/kg), LPS + FCPR03 (0.5 and 1 mg/kg, respectively), and rolipram (1 mg/kg) (*n* = 8 per group). Drugs were dissolved in 0.5% DMSO and 0.5% carboxymethylcellulose sodium, and were intragastrically administered for seven consecutive days. On days 7, 30 min after the last drug administration, saline or LPS were intraperitoneally injected in mice. Then, animals were executed by cervical dislocation after behavior tests, the brain tissue isolated and the hippocampus and cortex collected, stored at −80 °C for further biochemical analysis.

### 4.4. Sucrose Preference Test

Experiments were performed as previously described [34]. On the first day, mice were habituated to drinking from two bottles, both containing 5% sucrose solution. On the second day, one of the bottles was replaced with a bottle containing pure water. Mice were then deprived of water and food for 24 h prior to a 12-h testing session. Sucrose preference was defined as (weight of sucrose ingested)/(weight of water ingested + weight of sucrose ingested) × 100.

### 4.5. Open Field Test

OFT was used to measure spontaneous activity, since the forced swimming test evaluates a parameter that depends on locomotor activity, it is necessary to exclude confounding drug-inducing locomotor effects [32,34]. Mice were placed in a black wooden box (40 × 30 × 20 cm). Locomotion was recorded for 5 min using a camera, and analyzed using an open field experimental video analysis system (Smart 3.0, Panlab SMART video tracking system, Barcelona, Spain).

### 4.6. Forced Swim Test and Tail Suspension Test

FST and TST are another common behavioral test to evaluating depressive-like behavior in animals [32]. During the tests mice were placed in a clear glass cylinder (10-cm diameter, 25-cm height) containing fresh water at 25 ± 2 °C and suspended at 20 cm above the floor using adhesive tape placed 3 cm from the tip of the tail, testing lasted for 6 min, immobility time during the last 4 min was scored.

### 4.7. Assessment of Corticosterone Level in Plasma

CORT levels in plasma were determined by ELISA kit according to the manufacturer’s protocols. The CORT levels was normalized to total protein, and the samples were assayed in triplicate.

### 4.8. Western Blot Analysis

Western blotting was carried out according to previously published protocols, with slight modification [22]. Brain tissue were homogenized with the Radio Immunoprecipitation Assay (RIPA) lysis buffer (including 1% protease inhibitor cocktail, 1% phosphatase inhibitor cocktail) containing a protease inhibitor cocktail (Sigma-Aldrich), centrifuged at 12,000× *g* for 10 min, BCA protein assay kit (Thermo Scientific, Waltham, MA, USA) was used to measure the total protein concentration, and equal amounts of protein were separated by sodium dodecyl sulfate polyacrylamide gel electrophoresis. Protein bands were transferred to PVDF membranes, and incubated with a primary rabbit antibody against BDNF (1:1000; Cell Signaling Technology, Danvers, MA, USA), p-p38 (1:1000; Cell Signaling Technology, Danvers, MA, USA), p38 (1:1000; Cell Signaling Technology, Danvers, MA, USA), p-JNK (1:1000; Cell Signaling Technology, Danvers, MA, USA), JNK (1:1000; Cell Signaling Technology, Danvers, MA, USA), or rabbit GAPDH (1:10,000; Abcam, Cambridge, MA, USA) overnight at 4 °C, then incubated with goat anti-rabbit IgG horse radish peroxidase (1:5000; Cell Signaling Technology, Danvers, MA, USA) for 2 h at room temperature. The bands were quantified with Image J.

### 4.9. Statistical Analysis

All data are expressed as mean ± SEM, and differences were defined statistically significant only when *p* < 0.05. Data were analyzed by one-way ANOVA. All data were analyzed using the Statistical Package for the Social Sciences (SPSS) version 19.0 (SPSS Inc., Chicago, IL, USA). Figures were plotted using the GraphPad Prism 5.0 software (La Jolla, CA, USA).
